# Bluetongue in Eurasian Lynx

**DOI:** 10.3201/eid1409.080434

**Published:** 2008-09

**Authors:** Thierry P. Jauniaux, Kris E. De Clercq, Dominique E. Cassart, Seamus Kennedy, Frank E. Vandenbussche, Elise L. Vandemeulebroucke, Tine M. Vanbinst, Bart I. Verheyden, Nesya E. Goris, Freddy L. Coignoul

**Affiliations:** Author affiliations: Royal Belgian Institute of Natural Sciences, Brussels, Belgium (T.P. Jauniaux); University of Liege, Liege, Belgium (T.P. Jauniaux, D.E. Cassart, F.L. Coignoul); Veterinary and Agrochemical Research Centre, Brussels (K.E. De Clercq, F.E. Vandenbussche, E.L. Vandemeulebroucke, T.M. Vanbinst, B.I. Verheyden, N.E. Goris); Agri-Food and Biosciences Institute, Belfast, Northern Ireland, UK (S. Kennedy)

**Keywords:** bluetongue, carnivore, pathology, lynx, BTV, serotype 8, letter

**To the Editor:** Bluetongue is an infectious disease of ruminants; it is caused by bluetongue virus (BTV), has 24 known serotypes, and is transmitted by several species of *Culicoides* biting midges. The disease mainly affects sheep and occurs when susceptible animals are introduced to areas where BTV circulates or when BTV is introduced to naive ruminant populations. The natural host range is strictly limited to ruminants, although seroconversion without disease has been reported in carnivores (*1*). We report BTV infection, disease, and death in 2 Eurasian lynx (*Lynx lynx*) and the isolation of BTV serotype 8 (BTV-8) from this carnivorous species.

 The 2 Eurasian lynx, held in the same cage in a zoo in Belgium, became lethargic in September 2007; animal 1 died after 2 days, and animal 2 died in February 2008. Both had been fed ruminant fetuses and stillborns from surrounding farms in an area where many bluetongue cases had been confirmed (*2*). Necropsy findings for animal 1 were anemia, subcutaneous hematomas, petechial hemorrhages, and lung congestion with edema. Necropsy findings for animal 2 were emaciation, anemia, enlarged and gelatinous lymph nodes, petechial hemorrhages, and pneumonia. For each animal, microscopic examination showed edematous vascular walls; enlarged endothelial cells; and evidence of acute to subacute vasculitis in muscle, myocardium, peritoneum, and lung. Tissue samples (spleen, lung, intestine) were analyzed by using 2 real-time reverse transcriptase–quantitative PCR techniques targeting BTV segment 5 and host β-actin mRNA as a control. BTV RNA was found in all samples from animal 1; cycle threshold values (*3*) ranged from 28.6 to 36.2. Tissues from animal 2 were negative for BTV RNA. Although the internal control was originally designed to detect β-actin mRNA of bovine or ovine species, clear positive signals were noted in all lynx samples, which indicated that this was a reliable control procedure. Infectious virus was subsequently isolated from the lung sample of animal 1 after inoculation of embryonated chicken eggs and amplification in baby hamster kidney–21 cell cultures (*4*). The specificity of the cytopathic effect, observed 48 hours after passage on baby hamster kidney–21 cells, was confirmed by real-time reverse transcriptase–quantitative PCR. Virus neutralization using specific reference serum (*5*) proved that the isolated virus was BTV-8. Anti-BTV antibodies were detected in lung tissue fluid from animal 2 (ID Screen Bluetongue Competition assay, ID VET, Monpellier, France) (*6*).

We describe a natural, wild-type infection of a carnivorous species. Although deaths have been documented in dogs accidentally infected with a BTV-contaminated vaccine (*7*), the 2 lynx in this report were neither vaccinated nor medically treated by injection. BTV-8 was first introduced to northern Europe in 2006 and has subsequently spread rapidly to many countries on that continent. During 2007, a total of 6,870 bluetongue cases were reported in Belgium (*2*); animal 1 died in September 2007, which corresponded to the peak of bluetongue outbreaks in that region. No deaths were reported during that period among other animals, including ruminants, held in the same zoo as the 2 lynx reported here. The time lapse between initial clinical signs and death could explain the failure to detect BTV-8 RNA in animal 2. Although speculative, the suspicion of bluetongue in this animal is based on the presence of anti–BTV-8 antibodies, vasculitis, and pneumonia, which have been found in dogs accidentally infected with BTV (*7*).

This report raises questions about the current knowledge of the epidemiology of bluetongue. Bluetongue in lynx indicates that the list of known susceptible species must be widened, at least for serotype 8. Although infection of a susceptible host by an insect vector is the only proven natural transmission mechanism for wild-type BTV, transplacental transmission of BTV-8, resulting in the birth of seropositive (*8*) or virus-positive calves (*9*), has recently been described in cattle. Although infection by an insect vector cannot be excluded, transmission by the oral route must be strongly suspected because the lynx described in this report had been fed ruminant fetuses and stillborn animals from surrounding farms. This possibility is supported by a previous suspicion that seroconversion to BTV in carnivores was a result of oral infection (*1*). The possibility of oral transmission is also supported by evidence of lateral transmission of BTV infection to cattle having occurred, in the absence of insect vectors, as a result of direct contact with newborn viremic calves born to infected dams that had been imported to Northern Ireland from a bluetongue-infected region of continental Europe (S. Kennedy, unpub. data). The role of wildlife, especially carnivores, in the epidemiology of bluetongue deserves further study to elucidate their role as either dead-end hosts or new sources of infection for livestock and to help determine the risks for wildlife populations.

Our findings clearly indicate that a novel transmission pathway enables the virus to cross species. Consequently, transmission to other species, including domestic animals, can no longer be excluded. Moreover, oral transmission is likely to have considerable implications for disease control, including vaccination, because BTV-8 is a fast-emerging virus with major financial consequences.
